# Diabetes Is the Most Critical Risk Factor of Adverse Complications After Peritoneal Dialysis Catheter Placement

**DOI:** 10.3389/fmed.2021.719345

**Published:** 2021-10-27

**Authors:** Hsiao-Huang Chang, Ching-Hsiang Chang, Chen-Yuan Hsiao, Shih-Yi Kao, Jinn-Yang Chen, Tien-Hua Chen, Pei-Jiun Tsai

**Affiliations:** ^1^Division of Cardiovascular Surgery, Department of Surgery, Veterans General Hospital, Taipei, Taiwan; ^2^School of Medicine, College of Medicine, Taipei Medical University, Taipei, Taiwan; ^3^Nursing Department, Veterans General Hospital, Taipei, Taiwan; ^4^Division of Cardiovascular Surgery, Department of Surgery, Taipei Medical University Hospital, Taipei, Taiwan; ^5^Department of Biology and Anatomy, National Defense Medical Center, Taipei, Taiwan; ^6^Ten-Chan General Hospital Zhongli, Taoyuan, Taiwan; ^7^Division of Nephrology, Department of Medicine, Veterans General Hospital, Taipei, Taiwan; ^8^School of Medicine, Institute of Anatomy and Cell Biology, National Yang Ming Chiao Tung University, Taipei, Taiwan; ^9^Department of Surgery, Trauma Center, Veterans General Hospital, Taipei, Taiwan; ^10^Division of General Surgery, Department of Surgery, Veterans General Hospital, Taipei, Taiwan; ^11^Department of Critical Care Medicine, Veterans General Hospital, Taipei, Taiwan

**Keywords:** end-stage renal disease (ESRD), peritoneal dialysis, catheter, diabetes, infection

## Abstract

**Introduction:** Peritoneal dialysis (PD) is a kind of renal replacement therapy for end-stage renal disease (ESRD). While PD has many advantages, various complications may arise.

**Methods:** This retrospective study analyzed the complications of ESRD patients who received PD catheter implantation in a single medical center within 15 years.

**Results:** This study collected 707 patients. In the first 14 days after PD implantation, 54 patients experienced bleeding complications, while 47 patients experienced wound infection. Among all complications, catheter-related infections were the most common complication 14 days after PD implantation (incidence: 38.8%). A total of 323 patients experienced PD catheter removal, of which 162 patients were due to infection, while 96 were intentional due to kidney transplantation. Excluding those whose catheters were removed due to transplantation, the median survival of the PD catheter was 4.1 years; among them, patients without diabetes mellitus (DM) were 7.4 years and patients with DM were 2.5 years (*p* < 0.001). Further, 50% probability of surviving was beyond 3.5 years in DM patients with HbA1CC < 7 and 1.6 years in DM patients with HbA1C <7 (*p* ≥ 0.001).

**Conclusions:** Catheter-related infections were the most common complications following PD catheter implantation. DM, especially with HbA1C ≥7, significantly impacted on the catheter-related infection and the survival probability of the PD catheter.

## Introduction

End-stage renal disease (ESRD) is the final stage of chronic kidney disease (CKD). In the wake of economic development and progress in medical technology, the incidence and prevalence of ESRD worldwide have increased rapidly and continuously, making it a major challenge for the healthcare systems of many countries. Deterioration of renal function is irreversible in ESRD patients, resulting in not only the accumulation of metabolic waste and water in the body but also the imbalance of electrolyte and acid base. Appropriate renal replacement therapy may prevent the fatal condition and prolong the lives of ESRD patients. Hemodialysis (HD), peritoneal dialysis (PD), and kidney transplantation are the main types of renal replacement therapy. Though HD is previously the predominant type of dialysis in Taiwan, a steadily growing number of ESRD patients are opting to receive PD recently.

While the literature indicates that PD has many advantages, various complications after PD catheter implantation may arise, which are the chief concerns of patients who have decided to employ PD (1–3). The complications can be divided into two major categories of infective complications and non-infective complications (4). Infective complications include infections around the catheter exit site, subcutaneous tunnel infections, and peritonitis (5, 6). Of these, peritonitis is the most common complication of PD, which may increase the duration of hospitalization that even lead to death (7). Besides, severe or recurrent peritonitis is the chief factor of patients switching from PD to HD (8). The common pathogens of PD-related peritonitis include *Staphylococcus epidermidis, Staphylococcus aureus, Pseudomonas aeruginosa, Escherichia coli*, and *Salmonella* (9). Non-infective complications included increased intra-abdominal pressure that resulted in hernias, dialysate leakage, catheter obstruction, catheter malposition, and back pain (10), as well as metabolic change that resulted in bloody dialysate and chyloperitoneum (11).

The complications can also be divided into early and late complications, which referred to postoperative complications appearing within 14 days and those occurring after 14 days, separately (12). Early complications include abdominal pain, bleeding, and wound infection (13). Late complications include catheter-related infection, catheter malposition, catheter obstruction, dialysate leakage, hernia, bowel perforation, and encapsulating peritoneal sclerosis (EPS) (14). Catheter-related infections include peritonitis and infections at the catheter exit site or subcutaneous tunnel. Catheter malposition and catheter obstruction can block the ingoing and outgoing flow, which may result in further dialysate leakage, intestinal perforation, or hernia (15). EPS is the most severe complication arising from abdominal inflammation. Although rare, cases of EPS have an extremely poor prognosis and the mortality rate of EPS may exceed 70% (16).

This retrospective study recorded the type and incident rate of complications in ESRD patients receiving PD catheter implantation at a single medical center in Taiwan. The purpose of this study was to analyze the risk factors that affect the recorded complications. We hope that this study can provide a great reference to the medical team for better patient care. We also hope that the results of this study can help patients have more confidence in choosing PD as a renal replacement therapy.

## Materials and Methods

This study was a retrospective cohort study. The study was conducted in accordance with the Declaration of Helsinki, and the study protocol was approved by the Institutional Review Board (IRB) of Taipei Veterans General Hospital (approval number: 2015-05-007CC). The written informed consent from the participants for this retrospective study was waived by IRB.

**Figure 1:**
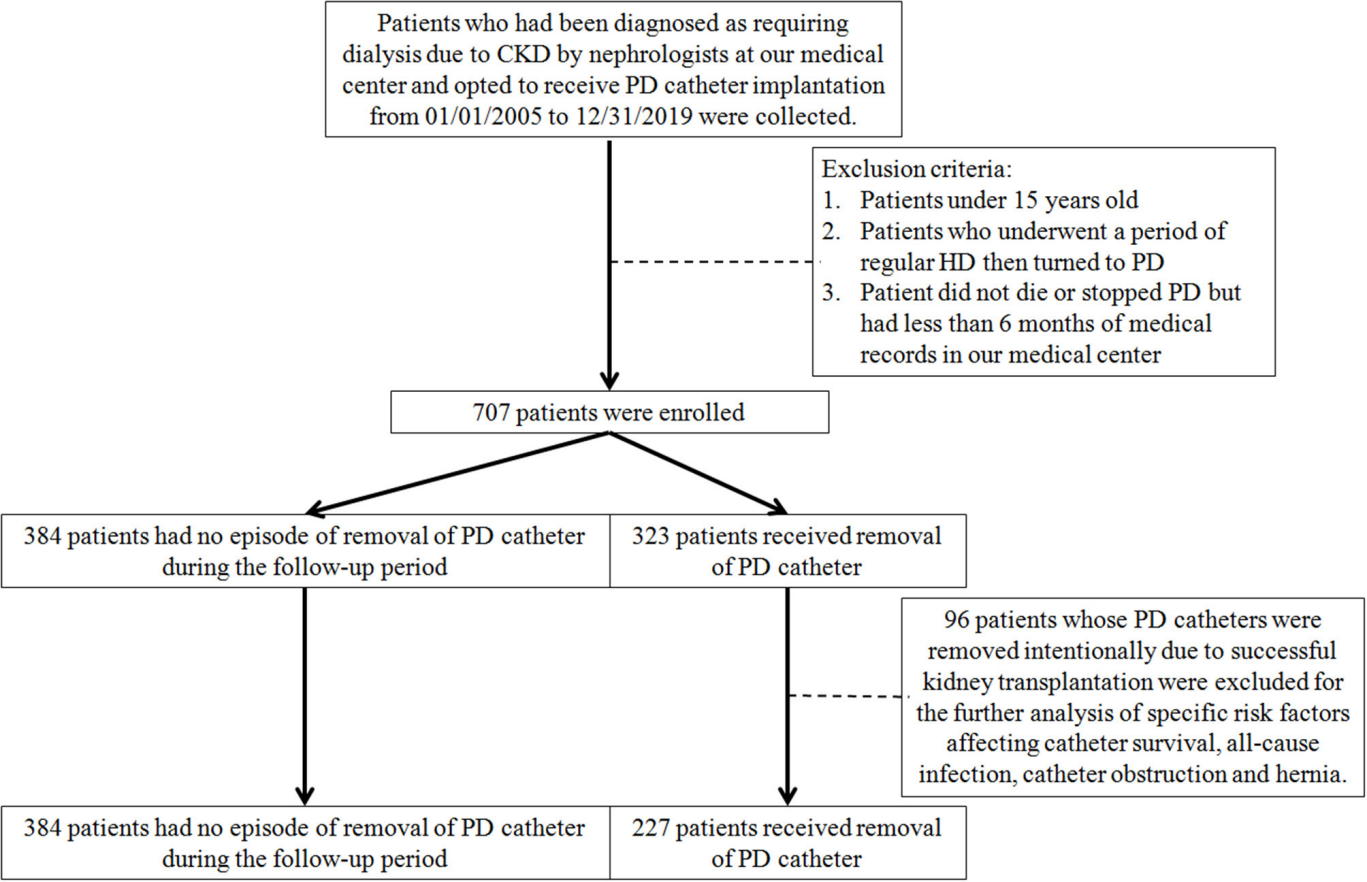
The flowchart for inclusion and exclusion of this study. CKD, chronic kidney disease; HD, hemodialysis; PD, peritoneal dialysis.

Patients who had been diagnosed as requiring dialysis due to CKD by nephrologists at the medical center and opted to receive PD catheter implantation from 01/01/2005 to 12/31/2019 were collected. All the clinical characteristics and the complications after PD implantations were recorded until stopping PD or 12/31/2020. Patients who picked and received regular PD at the beginning of renal replacement therapy were enrolled, while those underwent a period of regular HD and then turned to PD were excluded. Patients under 15 years old and those did not die or stopped PD but had <6 months of medical records in this medical center were also excluded. A flowchart of the inclusion and exclusion process is presented in Figure 1.

There was a dedicated PD team with a nephrologist, surgeon, ward nurse, case manager, pharmacist, dietitian, and social worker in our medical center. The case managers were usually full-time and specially trained PD nurses. Each PD patient has a dedicated case manager who was responsible for recording and managing the relevant medical information of the patient from the beginning of the PD catheter implantation operation. Once the CKD patient decided to receive PD, the case manager must help the nephrologists fill in the PD special chart and updated it regularly till the patient withdraw PD. In addition to the personal information, the PD special chart also recorded various clinically recognized variables related to the prognosis of renal failure and PD based on clinical knowledge. The past history and comorbidities from both patient self-reported and physician-reported were recorded.

All patients received laparoscopic implantation procedure with double-cuffed Tenckhoff PD catheter in our medical center (Baxter Healthcare Corporation. 21026 Alexander Couth, Hayward. CA 94545, United States). Prophylactic antibiotics were not used since the PD implantation procedure was a kind of clean-wound operation. After the PD catheter was inserted, the insertion site was usually covered with gauze dressing and tape to prevent the catheter from moving and keep the area clean and dry. The case manager would use sterile techniques to change the dressings of wounds and catheter exit site every catheter every day. Our PD team would arrange a comprehensive education program on PD catheter care and PD training for PD patients and their families. When the exit site healed, usually 2 weeks after surgery, PD treatment would begin.

All PD patients received regular follow-up for blood tests and imaging examinations at least once every 3 months in the nephrology clinic of our medical center. The case managers wound record all the relevant laboratory data on the PD special chart during the PD therapy period. Besides the usual daily medications for the comorbidities, the physician would prescribe medications to prevent or treat constipation. Complication of PD would also be recorded on the PD special chart.

In this study, we retrieved all the data from the PD special chart and performed further analysis. Glycated hemoglobin (HbA1C) would be check at least once every 3–6 months in diabetic patients while once a year in non-diabetic patients. During the PD treatment period, as long as there was one test report of HbA1C more than 7, the patient would be classified into the HbA1C ≥ 7 group. For patients in the HbA1C < 7 group, no HbA1C ≥7 event occurred during the PD treatment period.

Since patients that did not die or stopped PD but had <6 months of medical records in this medical center were excluded, there were no missing data in our study. This study employed SPSS 16.0 statistical software to perform analysis. Results were presented as mean±SD (standard deviation). Differences between groups were evaluated by Student's *t*-test or Mann–Whitney *U*-test for continuous variables and Fisher's exact test for categorical variables. All tests were two-tailed, and statistical significance was taken as *p* < 0.05. Survival curves of PD catheter were constructed using the Kaplan–Meier method, and differences were compared using the log-rank test. Cox proportional hazards model and multiple logistic regression model were used to figure out the significant risk factor of the different complications.

## Results

This study collected 707 patients, of which men accounted for 51.5% and women for 48.5%. The youngest ESRD patient receiving PD catheter implantation surgery was 17 years old, and the oldest was 87 years old; the mean age was 54 years old (54.07 ± 15.66), and ages had a normal distribution in the sample, with a median of 54 years old. Of the 707 patients, 97.2% had no prior abdominal surgery, but 2.8% did; the two most frequent types of prior abdominal surgery were appendectomy (*n* = 13) and cesarean section (*n* = 7). Table 1 summarizes the characteristics of the 707 patients.

**Table 1:** Clinical characteristics of the patients (*N* = 707).

	**Numbers**	**Percentage (%)**
Female	343	48.5
Male	364	51.5
**Age group (years)**		
<50	281	39.7
50–64	221	31.3
65–75	123	17.4
≥75	82	11.6
No history of abdominal surgery	687	97.2
History of abdominal surgery	20	2.8
**Chief cause of ESRD**		
Systemic lupus erythematosus	92	13.0
Diabetes mellitus	301	42.6
Chronic interstitial nephritis	96	13.6
Glomerulonephritis	100	14.1
Other causes	118	16.7
**Comorbidity**		
Hypertension	584	82.6
Diabetes mellitus	301	42.6
Coronary artery disease	144	20.4
Cerebrovascular accident	23	3.3
Respiratory disease	19	2.7
Hepatobiliary disease	80	11.3
**Number of comorbidities**		
0	60	8.5
1	281	39.7
2	247	34.9
3	103	14.6
4	16	2.3
**Catheter exit location**		
Left-Side abdomen	79	11.2
Right-Side abdomen	628	88.8
First catheter implantation	641	90.7
Catheter re-implantation	66	9.3
**Early complications (within 14 days after operation)**		
Bleeding	54	7.6
Wound infection	47	6.6
**Late complications (more than 14 days after operation)**		
Catheter-Related infectious complications	274	38.8
Catheter malposition	57	8.1
Catheter obstruction	27	3.8
Dialysate leakage	4	0.6
Hernia	90	12.7
Bowel perforation	0	0
Encapsulating peritoneal sclerosis	0	0
**Cause of removal of PD catheter (*****N*** **= 323)**		
Infection	162	50.2
Catheter obstruction	34	10.5
Transplantation	96	29.7
Other	31	9.6

*ESRD, end-stage renal disease; PD, peritoneal dialysis*.

End-stage renal disease has many causes. This study classified the cause of ESRD into five categories: systemic lupus erythematosus, diabetes mellitus (DM), chronic interstitial nephritis, glomerulonephritis, and other factors, including gout, hypertension, aristolochic acid nephropathy, IgA, IgM, and nephritic cystic kidney pathologies. Of the 707 patients, diabetes was the most common cause (42.6%), followed by other causes (16.7%), glomerulonephritis (14.1%), chronic interstitial nephritis (13.6%), and systemic lupus erythematosus (13.0%).

In this study, patients who had underlying disease of hypertension accounted for 82.6%, while those with DM accounted for 42.6%, those of coronary artery disease accounted for 20.4%, those of cerebrovascular accident accounted for 3.3%, those of respiratory disease accounted for 2.7%, and those of hepatobiliary disease accounted for 11.3%. Of the 707 patients, 2.3% had four kinds of the recorded comorbidities, 14.6% had three, 34.9% had two, 39.7% had one comorbidity, and 8.5% had no kinds of the recorded comorbidities.

The longest catheter implantation operation time was 120 min, the shortest was 20 min, and the mean length was 59.3 ± 10.6 min. The right side abdomen was the most common catheter exit location (88.8%), with the left side only 11.2%. A large majority of the patients received a first-time catheter implantation (90.7%), and re-implantation was performed in 66 patients (9.3%).

All the patients were followed up at the same medical center, and all the complications after PD implantations were recorded until stopping PD or 12/31/2020. The follow-up period ranged from 1 month to 14.16 years. The mean follow-up period was 5.48 years (5.48 ± 2.36), and the follow-up period had a normal distribution in the sample, with a median of 4.92 years. Wound bleeding, subcutaneous hematoma, and evidence of hemoperitoneum were recorded as postoperative bleeding complications. In the 707 patients, 54 patients (7.6%) experienced postoperative bleeding complications. The incidence rate of wound infection within 14 days after operation was 6.6% (47 patients). Catheter exit site infections, catheter tunnel infections, and peritonitis were all considered as catheter-related infectious complications. Among the complications that happened more than 14 days after the operation, the catheter-related infectious complications were most numerous and occurred in 38.8% (274 patients). In particular, peritonitis occurred in 246 patients (34.8%). The incidence of catheter malposition and catheter obstruction was 8.1% (57 patients) and 3.8% (27 patients) separately. Dialysate leakage occurred in 4 patients (0.6%). Postoperative hernia was the second most frequent late complication, which occurred in 90 patients (12.7%). The incidence of bowel perforation and EPS was 0.

About 323 patients (45.7%) encountered the removal of the PD catheter during the follow-up period. One hundred and sixty-two patients received the removal of PD catheter due to infection, 34 patients due to catheter obstruction, 96 patients due to successful kidney transplantation, 31 patients due to other causes, including the poor response of PD or other personal reasons. Considering the follow-up periods, the incidence rate of catheter removal was 8.34 cases per 100 patient-year. Excluding the patients whose catheters were removed intentionally due to successful kidney transplantation, the incidence rate of catheter removal was 6.67 cases per 100 patient-year.

After excluding patients whose catheters were removed intentionally due to successful kidney transplantation, the Cox proportional hazard model was used to analyze the specific risk factors affecting catheter survival (Table 2). Univariate analysis showed that age, comorbidity number, comorbidity with DM, and the HbA1C ≥7 status significantly made a contribution to the survival of the PD catheter. Further multivariate analysis was done and explored that age, comorbidity with DM, and the HbA1C ≥7 status indeed played a significantly important role in the removal of PD catheter.

**Table 2:** Proportional hazards model for PD catheter survival analysis.

	**Univariate analysis**			**Multivariate analysis**		
	**HR**	**95.0% CI**	***p*** **-value**	**HR**	**95.0% CI**	***p*** **-value**
Age	1.011	1.004 ~ 1.018	<0.001*	1.011	1.006 ~ 1.021	<0.001*
Gender-Male	Ref.			Ref.		
Gender-Female	0.829	0.665 ~ 1.032	0.094	0.888	0.707 ~ 1.116	0.310
Site-Right	Ref.			Ref.		
Site-Left	1.176	0.850 ~ 1.627	0.327	1.175	0.838 ~ 1.649	0.350
Abdominal operation history	0.518	0.214 ~ 1.253	0.144	0.538	0.217 ~ 1.335	0.181
Comorbidity: hypertension	1.348	0.986 ~ 1.842	0.061	1.042	0.748 ~ 1.451	0.810
Comorbidity: diabetes mellitus	2.231	1.784 ~ 2.791	<0.001*	2.333	1.624 ~ 3.351	<0.001*
Comorbidity: coronary heart disease	0.824	0.620 ~ 1.095	0.181	0.692	0.475 ~ 1.009	0.056
Comorbidity: cerebrovascular accident	0.652	0.291 ~ 1.463	0.299	0.671	0.295 ~ 1.526	0.342
Comorbidity: respiratory disease	0.721	0.371 ~ 1.403	0.336	0.904	0.454 ~ 1.798	0.773
Comorbidity: hepatobiliary disease	0.797	0.553 ~ 1.149	0.224	1.003	0.683 ~ 1.472	0.694
Comorbidity number	1.189	1.057 ~ 1.337	0.004*	1.171	0.998 ~ 1.374	0.053
HbA1C < 7	Ref.			Ref.		
HbA1C ≥7	3.606	2.726 ~ 4.771	<0.001*	2.959	1.997 ~ 4.385	<0.001*
Cause of ESRD-Systemic lupus erythematosus	Ref.			Ref.		
Cause of ESRD-Diabetes mellitus	1.246	0.782 ~ 1.985	0.354	1.156	0.719 ~ 1.860	0.549
Cause of ESRD-Chronic interstitial nephritis	0.983	0.581 ~ 1.663	0.948	0.861	0.505 ~ 1.468	0.583
Cause of ESRD-Glomerulonephritis	0.641	0.391 ~ 1.049	0.077	0.595	0.360 ~ 0.983	0.053
Cause of ESRD-Other	0.413	0.261 ~ 0.954	0.061	0.000	0.253 ~ 0.944	0.091

*PD, peritoneal dialysis; HR, Hazards ratio; CI, confidence interval; HbA1C, Glycated hemoglobin, hemoglobin A1C; ESRD, end-stage renal disease*.

* *p < 0.05*.

**Figure 2:**
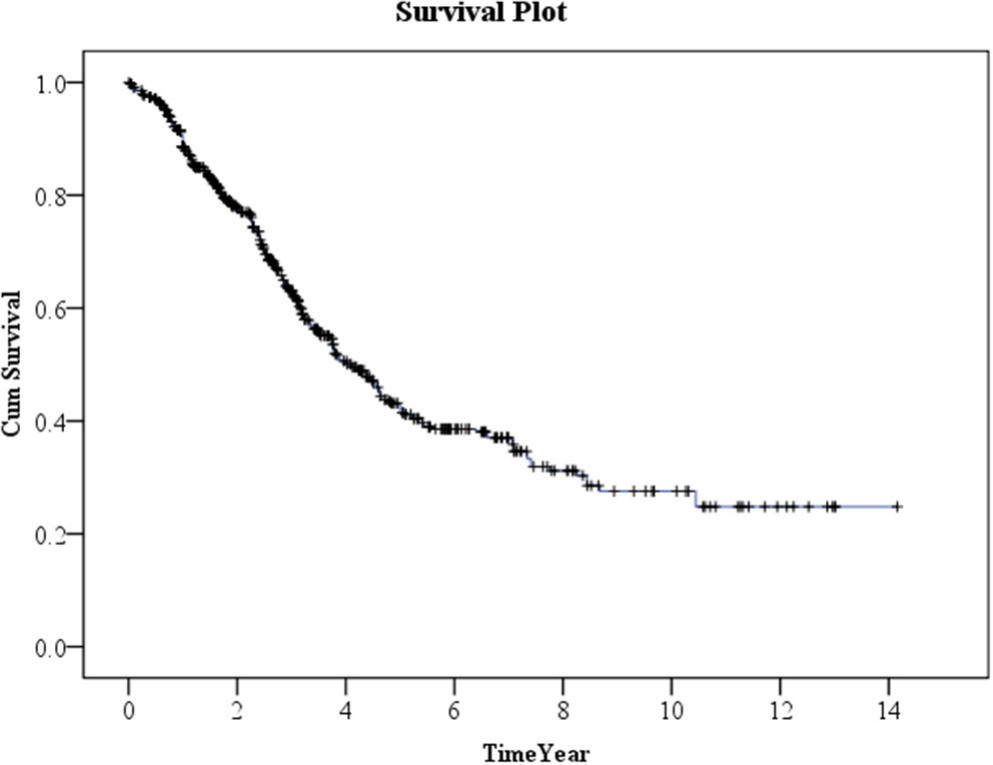
The survival pot of PD catheter in all patients. PD, peritoneal dialysis.

**Figure 3:**
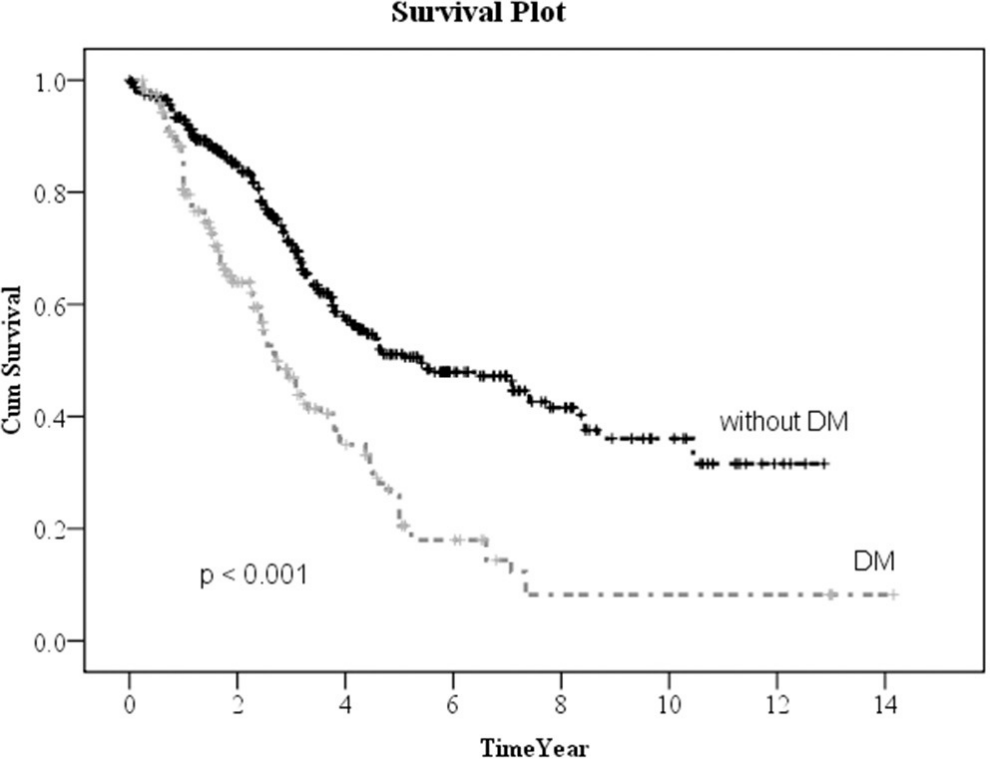
The survival pot of PD catheter in patients with and without diabetes. PD, peritoneal dialysis.

**Figure 4:**
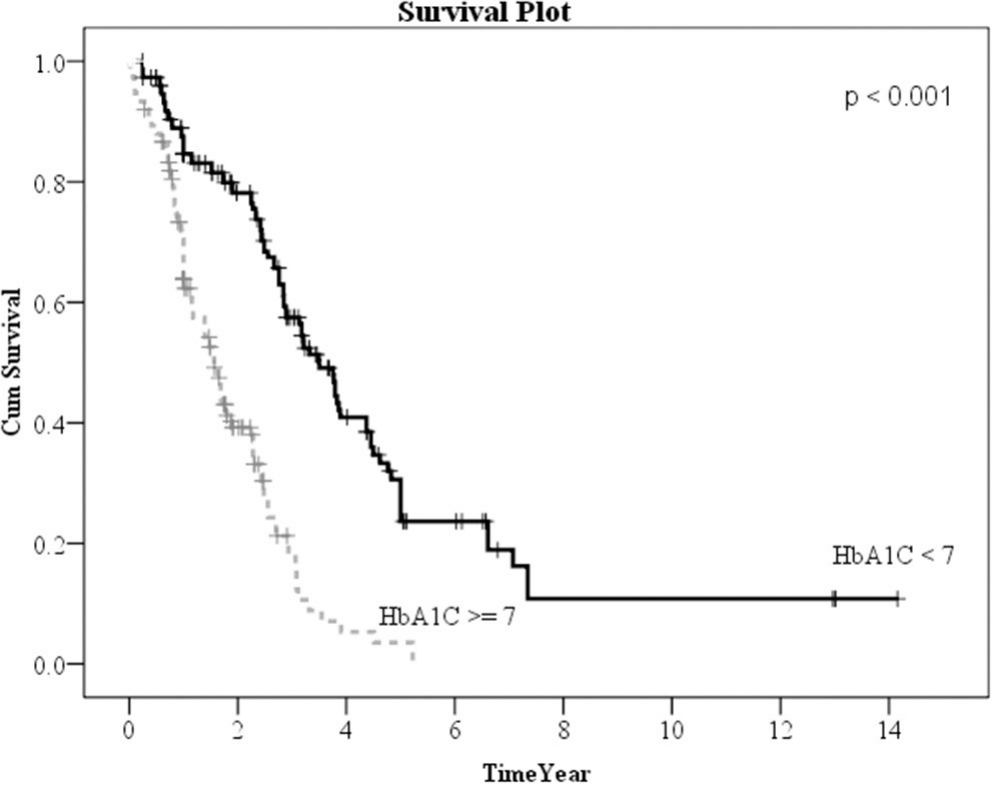
The survival pot of PD catheters in diabetes patients with HbA1C < 7 and ≥7. PD, peritoneal dialysis.

**Figure 5:**
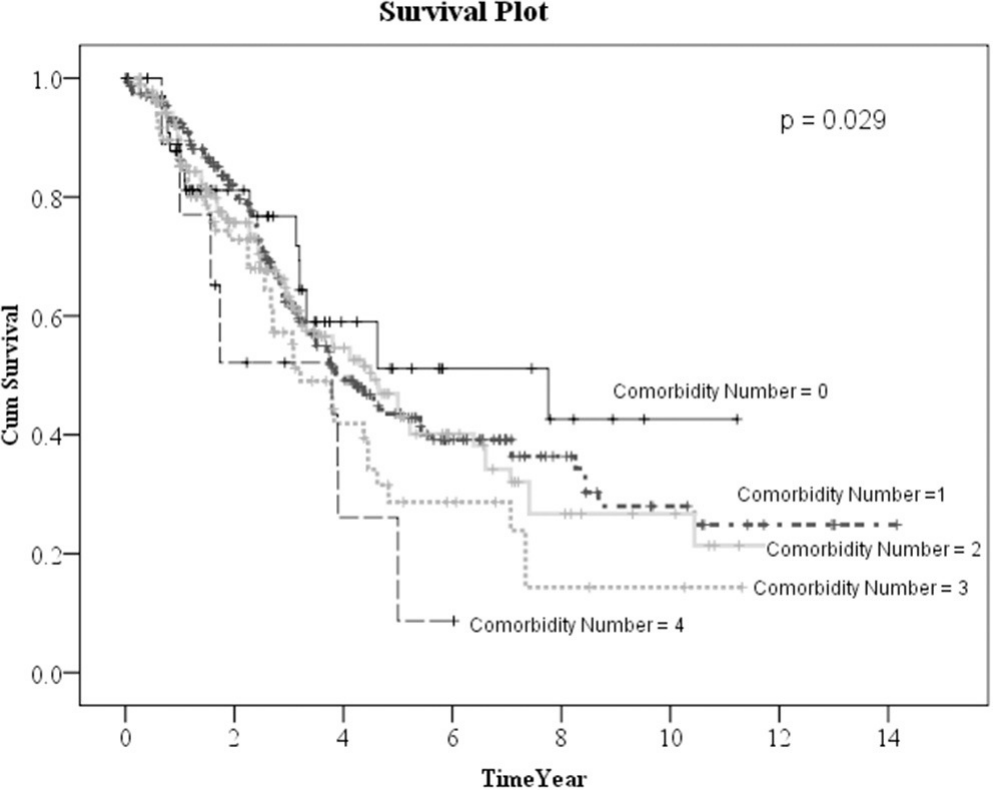
The survival pot of PD catheters in all patients with different comorbidity number. PD, peritoneal dialysis.

Kaplan–Meier method was used to calculate the survival probability of the PD catheter. The median survival of the PD catheter was 4.1 years in all patients (Figure 2). When we compared the patients without and with DM, we found that the median survival of the PD catheter was 7.4 years in patients without DM and 2.5 years in patients with DM (*p* < 0.001; Figure 3). Further, 50% probability of surviving was beyond 3.5 years in DM patients with HbA1C < 7 and 1.6 years in DM patients with HbA1C ≥7 (*p* < 0.001; Figure 4). Kaplan–Meier survival plot and log-rank test were also done to analyze the impact of comorbidity number on the PD catheter survival, which showed that the more comorbidity number, the lower PD catheter survival time (Figure 5).

Finally, we used the multiple logistic regression method to analyze the factors influencing all-cause infection (Table 3), catheter obstruction, and hernia (Table 4) after catheter implantation, respectively. DM, especially with HbA1C ≥7, significantly impacted on the all-cause infection. Left-side catheter exit location, abdominal operation history, and DM comorbidity significantly increased the occurrence of catheter obstruction. Compared with women, men were more likely to have hernias after PD catheter implantation.

**Table 3:** Multiple logistic regression analysis of factors influencing infection after catheter implantation.

	**Adjusted OR**	**95.0% CI**	***p*** **-value**
Age	1.020	0.989 ~ 1.040	0.263
Gender-Male		Ref.	
Gender-Female	0.781	0.559 ~ 1.092	0.148
Site-Right		Ref.	
Site-Left	0.840	0.498 ~ 1.428	0.520
Abdominal operation history	0.308	0.066 ~ 1.440	0.135
Comorbidity: hypertension	0.866	0.550 ~ 1.364	0.536
Comorbidity: diabetes mellitus	2.859	0.911 ~ 3.771	0.003 *
Comorbidity: coronary heart disease	0.814	0.525 ~ 1.264	0.360
Comorbidity: cerebrovascular accident	0.744	0.289 ~ 1.918	0.541
Comorbidity: respiratory disease	1.664	0.600 ~ 4.613	0.328
Comorbidity: hepatobiliary disease	0.515	0.291 ~ 1.012	0.073
Comorbidity number	1.032	0.729 ~ 1.190	0.572
HbA1C < 7		Ref.	
HbA1C ≥ 7	3.808	2.177 ~ 6.662	< 0.001 *
Cause of ESRD-Systemic lupus erythematosus		Ref.	
Cause of ESRD-Diabetes mellitus	2.597	1.202 ~ 5.612	0.015 *
Cause of ESRD-Chronic interstitial nephritis	2.197	0.911 ~ 5.295	0.080
Cause of ESRD-Glomerulonephritis	1.594	0.734 ~ 3.461	0.238
Cause of ESRD-Other	0.825	0.401 ~ 1.698	0.602

*OR, Odds ratio; CI, confidence interval; HbA1C, Glycated hemoglobin, hemoglobin A1C; ESRD, end-stage renal disease*.

* *p < 0.05*.

**Table 4:** Multiple logistic regression analysis of factors influencing catheter obstruction and hernia after implantation.

	**Catheter obstruction**			**Hernia**		
	**Adjusted OR**	**95.0% CI**	***p*** **-value**	**Adjusted OR**	**95.0% CI**	***p*** **-value**
Age	1.000	0.973 ~ 1.027	0.983	1.001	0.986 ~ 1.015	0.916
Gender-Male		Ref.			Ref.	
Gender-Female	0.605	0.263 ~ 1.394	0.238	0.411	0.252 ~ 0.671	<0.001*
Site-Right		Ref.			Ref.	
Site-Left	3.323	1.275 ~ 8.663	0.014*	1.494	0.766 ~ 2.915	0.238
Abdominal operation history	7.560	1.656 ~ 34.515	0.009*	0	0	0.999
Comorbidity: hypertension	1.999	0.434 ~ 9.214	0.374	1.049	0.560 ~ 1.965	0.880
Comorbidity: diabetes mellitus	4.355	1.261 ~ 15.042	0.020*	0.786	0.364 ~ 1.697	0.540
Comorbidity: coronary heart disease	0.856	0.332 ~ 2.207	0.748	0.690	0.361 ~ 1.318	0.261
Comorbidity: cerebrovascular accident	0.000	0.000	0.998	1.116	0.303 ~ 4.112	0.869
Comorbidity: respiratory disease	1.672	0.196 ~ 14.240	0.638	1.021	0.218 ~ 4.786	0.979
Comorbidity: hepatobiliary disease	0.773	0.171 ~ 3.489	0.738	0.965	0.474 ~ 1.965	0.921
Comorbidity number	1.125	0.631 ~ 2.004	0.691	1.089	0.781 ~ 1.520	0.615
HbA1C <7		Ref.			Ref.	
HbA1C ≥ 7	1.408	0.484 ~ 4.098	0.529	0.553	0.230 ~ 1.331	0.186
Cause of ESRD-Systemic lupus erythematosus		Ref.			Ref.	
Cause of ESRD-Diabetes mellitus	1.648	0.152 ~ 17.911	0.682	1.314	0.412 ~ 4.189	0.645
Cause of ESRD-Chronic interstitial nephritis	2.570	0.212 ~ 31.180	0.459	0.668	0.153 ~ 2.910	0.591
Cause of ESRD-Glomerulonephritis	3.299	0.329 ~ 33.105	0.310	2.185	0.696 ~ 6.866	0.181
Cause of ESRD-other	2.199	0.236 ~ 20.447	0.489	1.155	0.382 ~ 3.491	0.798

*OR, Odds ratio; CI, confidence interval; HbA1C, Glycated hemoglobin, hemoglobin A1C; ESRD, end-stage renal disease*.

* *p < 0.05*.

## Discussions and Conclusions

This study involved a 16-year retrospective investigation of the incidence of PD catheter implantation-related complications at a certain medical center. The results revealed that PD catheter-related infections were the most frequent complication (38.8%). This result was compatible with those at other large medical centers in the literature (17–19).

Catheter exit site infections and peritonitis were reported to be the main types of infection complication after PD catheter implantation (20). Catheter exit site infections may further lead to tunnel infection or peritonitis and would increase the length of hospital stay and PD failure rate (21). Generally, the factors associated with exit site infection included catheter type, catheter exit location, the existence of a hematoma at the exit site, diabetes, and obesity (22). The risk factors for peritonitis that had been reported include advanced age, female gender, indigenous racial origin, black ethnicity, low socioeconomic status, diabetes, cardiovascular disease, chronic lung disease, hypertension, and poor residual kidney function, obesity, smoking, living far from the PD hospital, depression, hypoalbuminemia, hypokalemia, absence of vitamin D supplementation, use of biocompatibility dialysate, nasal *S. aureus*, any previous exit-site infections, pets at home, and inadequate patient training (20–23).

In our result, 323 patients (45.7%) encountered removal of the PD catheter during the follow-up period. The majority (50.2%) of the patients who received the removal of the PD catheter were due to infection. After excluding patients whose catheters were removed intentionally due to successful kidney transplantation, we used the multiple logistic regression method to analyze the factors influencing all-cause infection. Only DM, especially with HbA1C ≥ 7, significantly impacted on the all-cause infection. Besides, we defined the event of catheter removal as expiry of the catheter. For the patients whose catheters were removed not intentionally due to transplantation, Kaplan–Meier survival plot illustrated that DM, especially with HbA1C ≥ 7, had a great impact on the survival probability of the PD catheter. The median survival of the PD catheter was 7.4 years in patients without DM and 2.5 years in patients with DM (*p* < 0.001). Fifty percentage probability of surviving was beyond 3.5 years in DM patients with HbA1C < 7 and 1.6 years in DM patients with HbA1C ≥ 7 (*p* < 0.001). Literature has indicated that due to vascular diseases, peripheral neuropathy, and other factors, the lack of blood supply in diabetic patients may further lead to insufficient immunity. Some pathogens tend to multiply rapidly in the hyperglycemic body. Therefore, diabetic patients were particularly susceptible to complications caused by infection after surgery and can easily become serious (24).

In this study, patients over the age of 75 accounted for 11.6% of the sample (82 patients), and the oldest patient was 87 years. There was no significant correlation with age and the complication rate after PD implantation, which implied that PD catheter implantation surgery was suitable for patients in all age groups, even elderly patients. This finding differed from those of certain international studies that found that age was an independent risk factor for infective complication (25, 26). This discrepancy may be due to the fact that our medical center was built specifically for elderly veterans and we had extensive experience in providing postoperative care to older patients. Besides the particularity of the hospital setting, the more important point was that we had a comprehensive medical care team to take care of PD patients, including physicians, surgeons, nurses, nutritionists, social workers, and rehabilitation specialists. PD catheters were crucial for patients choosing to receive PD as a renal replacement therapy. Practical experience had revealed that the issue of most concern to patients and their family members was how to avoid complications after catheter implantation surgery, and to maintain normal catheter function. A rate of complications of zero will be one of the greatest hopes of PD patients, and a goal toward which our medical team is working. In addition to carefully assessing the patient's living conditions before surgery, giving appropriate health education, including thoroughgoing knowledge and familiarity with residential care techniques, regular visits and evaluation of the patient's residential environment, living habits, and diet after surgery, are also very important. Our results showed that as long as the patient has HbA1C ≥ 7 during PD, it would significantly affect the survival rate of PD catheters. Therefore, if blood glucose can be strictly controlled during PD and HbA1C can be controlled below 7, the survival time of PD catheters can be prolonged and patients can obtain longer PD treatment time. Thus, our PD team must be more committed to strengthening blood sugar control of the PD patients and the associated perioperative health education in the future.

We also used the multiple logistic regression method to analyze the factors influencing catheter obstruction and hernia after catheter implantation, respectively. The redundant sigmoid colon may lead to more catheter obstruction rate for the left-side catheter exit location surgery. Besides, intra-abdominal adhesion after a previous abdominal operation may also significantly increased the occurrence of catheter obstruction. In PD treatment, a large amount of dialysate must be infused into the abdominal cavity every day, which may cause elevated abdominal pressure and then lead to the presence of hernias (including inguinal hernia or umbilical hernia). Of course, compared with women, men were more likely to have hernias after PD catheter implantation. Regardless of whether patients on PD present as single or bilateral inguinal hernias, we recommended that patients received bilateral inguinal hernioplasty.

In PD therapy, EPS was a late-stage and relatively severe complication. EPS was caused by repeated multifactor peritonitis and improper treatment, and often resulted in death by delaying the time of extubation. The incidence of EPS and death rate from EPS typically peaked around 7 years after catheter implantation (27, 28). According to the literature, an Australian study reported that the incidence of EPS was 19.4% after 8 years of PD treatment (27), while a Japan study reported as 2.1% after 8 years, 5.9% after 10 years, and 17.2% after 15 years, respectively (28). In our study, the incidence of EPS was zero. This may be because our PD medical team never hesitate to perform extubation when PD complications occurred, thus avoiding the occurrence of EPS.

In our study, patients received catheter re-implantation because the original catheter had to be removed due to complications such as infection, dislocation, and congestion. Generally speaking, catheter re-implantation may involve greater technical difficulty than initial catheter implantation. Peritoneal adhesions and changes in anatomical location may increase the chance of perioperative bleeding and visceral organ injury (29, 30). Intra-abdominal adhesions after previous abdominal surgery, with an incidence of about 60–80%, were traditionally considered to be related to a higher PD failure rate. However, our results, in fact, showed no higher incidence of complications associated with receiving catheter re-implantation. Therefore, we emphasized that when the medical team judged that the patient has to remove the PD catheter, he should not hesitate to remove the PD catheter, especially if not doing so may cause serious sequelae or even death. The medical team did not have to worry about re-implanting the catheter that would increase the risk of complications.

This study has limitations, which would inevitably affect the interpretation of these results. First, this was a single-center observational study. Besides, the variables of this study were only those clinically recognized variables related to the prognosis of renal failure and PD based on clinical knowledge recorded on the previously designed PD special chart, which may cause a certain degree of selection bias in the statistical results. However, it was worth noting that the results presented here were consistent with the experience of other medical centers in the world. It was also important that if we collected data from the inconsistent medical records of different medical centers, there may be many missing data that may lead to limitations in the interpretation of the results.

In conclusion, this was a retrospective cohort study including a total of 707 patients. Catheter-related infections were the most common postoperative complications following PD catheter implantation. DM, especially with HbA1C ≥ 7, significantly impacted on the catheter-related infection as well as the survival probability of the PD catheter. As a consequence, when caring for patients receiving PD catheter implantation, medical teams should strengthen their health education on the blood sugar control perioperatively, to help these patients reduce their likelihood of catheter-related infective complications.

## Data Availability Statement

The original contributions presented in the study are included in the article/supplementary material, further inquiries can be directed to the corresponding author/s.

## Ethics Statement

The study protocol was approved by the Institutional Review Board (IRB) of Taipei Veterans General Hospital (approval number: 2015-05-007CC). Written informed consent for participation was not required for this study in accordance with the national legislation and the institutional requirements.

## Author Contributions

H-HC, T-HC, and P-JT involved in conceptualization and funding acquisition. J-YC, C-HC, and P-JT involved in data curation and formal analysis. C-HC, C-YH, S-YK, and J-YC involved in investigation. H-HC and T-HC involved in methodology and project administration. T-HC and P-JT writing the original draft. H-HC, C-HC, C-YH, S-YK, J-YC, T-HC, and P-JT involved in writing the review and editing. All authors read and approved the final manuscript.

## Funding

This study was sponsored by grants from Taipei Veterans General Hospital (V108C-044, V109C-130, V108B-002, and V109C-131), Ten-Chan General Hospital Zhongli, Ministry of Science and Technology (MOST 108-2314-B-010-053 and MOST 107-2314-B-010-056-MY3), and Taiwan Association of Cardiovascular Surgery Research. The funding sources had no role in the study design, data collection, data interpretation, data analysis, or writing of the report.

## Conflict of Interest

The authors declare that the research was conducted in the absence of any commercial or financial relationships that could be construed as a potential conflict of interest.

## Publisher's Note

All claims expressed in this article are solely those of the authors and do not necessarily represent those of their affiliated organizations, or those of the publisher, the editors and the reviewers. Any product that may be evaluated in this article, or claim that may be made by its manufacturer, is not guaranteed or endorsed by the publisher.
